# Identification of Factors Influencing the Adoption of Health Information Technology by Nurses Who Are Digitally Lagging: In-Depth Interview Study

**DOI:** 10.2196/15630

**Published:** 2020-08-14

**Authors:** Jacqueline A De Leeuw, Hetty Woltjer, Rudolf B Kool

**Affiliations:** 1 Department of Information Management Radboud University Medical Center Nijmegen Netherlands; 2 Unit Process Improvement and Implementation Radboud University Medical Center Nijmegen Netherlands; 3 Department IQ Healthcare Institute for Health Sciences Radboud University Medical Center Nijmegen Netherlands

**Keywords:** qualitative research, semi-structured interview, purposive sampling, health information systems, computer user training, professional education, professional competence, registered nurses, nursing informatics

## Abstract

**Background:**

The introduction of health information technology (HIT) has drastically changed health care organizations and the way health care professionals work. Some health care professionals have trouble coping efficiently with the demands of HIT and the personal and professional changes it requires. Lagging in digital knowledge and skills hampers health care professionals from adhering to professional standards regarding the use of HIT and may cause professional performance problems, especially in the older professional population. It is important to gain more insight into the reasons and motivations behind the technology issues experienced by these professionals, as well as to explore what could be done to solve them.

**Objective:**

Our primary research objective was to identify factors that influence the adoption of HIT in a sample of nurses who describe themselves as digitally lagging behind the majority of their colleagues in their workplaces. Furthermore, we aimed to formulate recommendations for practice and leadership on how to help and guide these nurses through ongoing digital transformations in their health care work settings.

**Methods:**

In a Dutch university medical center, 10 face-to-face semi-structured interviews were performed with registered nurses (RN). Ammenwerth’s FITT-framework (fit between the Individual, Task, and Technology) was used to guide the interview topic list and to formulate themes to explore. Thematic analysis was used to analyze the interview data. The FITT-framework was also used to further interpret and clarify the interview findings.

**Results:**

Analyses of the interview data uncovered 5 main categories and 12 subthemes. The main categories were: (1) experience with digital working, (2) perception and meaning, (3) barriers, (4) facilitators, and (5) future perspectives. All participants used electronic devices and digital systems, including the electronic health record. The latter was experienced by some as user-unfriendly, time-consuming, and not supportive in daily professional practice. Most of the interviewees described digital working as “no fun at all,” “working in a fake world,” “stressful,” and “annoying.” There was a lack of general digital knowledge and little or no formal basic digital training or education. A negative attitude toward computer use and a lack of digital skills contributed to feelings of increased incompetency and postponement or avoidance of the use of HIT, both privately and professionally. Learning conditions of digital training and education did not meet personal learning needs and learning styles. A positive impact was seen in the work environment when colleagues and nurse managers were aware and sensitive to the difficulties participants experienced in developing digital skills, and when there was continuous training on the job and peer support from digitally savvy colleagues. The availability of a digital play environment combined with learning on the job and support of knowledgeable peers was experienced as helpful and motivating by participants.

**Conclusions:**

Nurses who are digitally lagging often have had insufficient and ineffective digital education. This leads to stress, frustration, feelings of incompetency, and postponement or avoidance of HIT use. A digital training approach tailored to the learning needs and styles of these nurses is needed, as well as an on-the-job training structure and adequate peer support. Hospital management and nurse leadership should be informed about the importance of the fit between technology, task, and the individual for adequate adoption of HIT.

## Introduction

### Background

Health care has been rapidly transformed by the introduction of health information technology (HIT) [1-3]. The introduction of the electronic health record (EHR) and different eHealth devices have drastically changed the daily practice of health care professionals and the way that health care is delivered. This change will continue as robots and artificial intelligence become gradually embedded into health care [4-6]. It is generally believed that HIT adds to the safety and quality of health care and reduces morbidity and mortality. This requires broad adoption, implementation, and other changes in health care processes and structures, on an individual, national, and organizational level [7,8].

Several barriers have been identified in the implementation of HIT and the associated changes impacting different organizational levels, such as the structure of the organization, the tasks performed, the incentives given, and the way information processes are developed and organized [8,9]. The adoption of digital technology is a complex process with several influencing factors on the individual level, such as perceived ease of use and usefulness, training, helping conditions, and personality traits, as well as computer anxiety and self-efficacy [10-12]. Negative and positive emotions influence the learning process and must be acknowledged during training [13]. As such, the uptake of HIT by health professionals does not always match expectations [14,15]. There is a growing awareness and general acceptance of the need for a sociotechnical approach to the implementation of HIT, emphasizing the importance of focusing on the social aspects of HIT implementation as well as on the technical aspects of a system. The implementation process is far from linear and predictable, and the fit between work processes and tasks, information technology, and individual characteristics determines the success of implementation [9].

In the Netherlands, nurses are the largest group of registered health professionals, and the government is strongly stimulating HIT in all health care settings [7]. As such, it follows that nurses will be increasingly confronted with and involved in HIT developments. Research shows several affecting factors in the general population of nurses, such as the fact that little attention has been given to the influence of HIT on the workflow of nurses in the early stages of HIT implementation, and a lack of digital training and organizational support [16,17]. Currently, there is little research on the impact of HIT on the daily work of nurses, although it has been suggested that it has the potential to reduce health care costs and improve quality of care [18]. Until now, there have not been specific studies addressing the target group of nurses who are digitally lagging behind the majority of working nurses in the field. A literature review study [19] on issues and concerns related to the adoption and use of electronic medical records (EMR) reveals the importance of the consideration and exploration of attitudes in nurses and their personal use of information technology (IT) devices regarding EHR adoption. This study also shows that negative attitudes toward computer usage, minimal skill levels, and low levels of change readiness do not improve self-confidence in nurses regarding IT adoption. This combination of factors is more likely to be present in older nurses, suggesting that this group might need more training than others who are more comfortable around computers. Hence, the commonly applied one-size-fits-all training approach might not be effective in this situation. Another study [20], using self-assessment scales to measure computer literacy and attitudes towards computer use in registered nurses, found that 1-3% of participant scores (N=688) fell into categories representing inadequate digital skills or cyberphobia. The age of the participants in these categories was not described. Some age-correlated results were found in a study [21] with an intention-to-use survey in a group of 113 registered nurses, revealing that older nurses (ages not defined) had statistically significantly less perceived computer self-efficacy than their younger colleagues.

The aging workforce of nursing has significant implications for the near future of nursing care and health care in general, especially in relation to increasing nursing shortages [22-24]. Not only do older nurses struggle with the physical demands of nursing work, but cognitive declines are also becoming more manifest, exemplified in struggles to keep pace with paper and digital work and dealing with declines in memory. These aspects could be exacerbated by the ever-changing workplace, the speed of technological advances, and the need to continuously develop new skills. Coping with many professional demands and changes is more challenging at an older age [23,25,26]. Hence, age is viewed as a predisposing factor. Health care organizations are challenged with creating healthy working environments that stimulate change readiness and motivate nurses of all ages to continue working and become competent and digitally skilled health professionals [22,27].

In the past two decades, several studies have looked at the experiences of nurses confronted with the demands of a rapidly evolving digital health care environment [21,27-30]. Several of these studies focused on nurses’ experiences with EHR implementation [21,28-30]. Research methods varied, including individual interviews, focus groups, surveys, and observations in daily practice. When individual interviews were performed, the scope was on EHR usability and adoption. One study [28] examined EHR adoption by health care professionals by studying the way they make sense of HIT. A general finding of all these studies was that the adoption and implementation of HIT needs thorough insight and knowledge of the way different groups of health care professionals handle substantial changes in their work routines. A systematic review on the implementation of EHR in hospitals identified that organizations and their leadership should be alert to managing the balance between the technology and the work processes to improve the fit between health care professionals and the HIT they use [9].

### Study Aim

There is a growing number of studies that report on the factors that influence HIT adoption in health care professionals. However, we specifically wanted to explore the experiences and needs of nurses who define themselves as digitally lagging behind the majority of their colleagues at their workplaces. This is a small and sometimes invisible group of professionals who are known to struggle with the demands of digital transformation in health care. Our aim was to uncover the views and needs of these nurses, and to deepen our understanding of the contextual and individual characteristics that affect their situation. Furthermore, we wanted to formulate recommendations for practice and leadership on how to help and guide these nurses through ongoing digital transformations in their health care work setting. We aimed to identify factors that influence the adoption of HIT in a sample of nurses who describe themselves as digitally lagging behind the majority of their colleagues at their workplaces.

## Methods

### Setting

The study was conducted in Radboud University Medical Center, a Dutch university hospital setting with 600 inpatient beds. In 2013, a new and fully integrated EHR was implemented hospital-wide. This hospital is one of the leading digital front runners in the Netherlands, and it is accredited with a HIMSS/EMRAM stage 7 status since 2015.

### Data Collection

From November to December 2017, semi-structured interviews were conducted with 10 registered nurses. Participants were selected by purposive sampling. In August 2017, an email was sent to all nurse managers of the hospital departments asking whether they knew nurses who had difficulties working with HIT in daily practice. The nurse managers drew from information gained from regular performance interviews conducted between nurse management and individual nurse staff members, in which usage of HIT, technology, and software systems is a standard item. The nurse managers identified the nurses who met our criteria and asked them to consider taking part in our research. Those who expressed interest were contacted and informed of the details of the research and the interview. Of the nurses who were approached, 10 agreed to an interview and were included after providing written consent for study participation.

### Interviews

Prior to the interview, nurses were sent an interview topic list so they could prepare themselves. Of the 10 interviews that were conducted, 9 occurred in a location outside of the hospital. The trained interviewer had no professional or private relationship with any of the participants. The interviewer made field notes during the interviews. After completing 2 interviews, the integral audio recordings of the interviews were listened to, and the topic list and interview techniques were confirmed. No further adjustments to the topic list were considered necessary. The mean duration of the interviews was 84 minutes (65 minutes minimum, 103 minutes maximum). All interviews were audio-recorded and transcribed. The manuscripts were sent to the participants for member checks and triangulation. One respondent wanted some changes made to the interview manuscript.

### Interview Topic List

We identified possible themes in the current literature to explore during the interviews. A concept topic list was made, and we decided to use Ammenwerth’s FITT-framework [31] as a basis for further development of the interviews. We compared our concept topic list with the different fit-axes of the framework to see if they were all broadly covered (Table 1). In addition, we discussed the topic list with several experts on information and communications technology (ICT) implementation and change management in the hospital.

**Table 1:** Interview topic list compared with the FITT-framework fit-axes; FITT: fit between Individual, Task, and Technology.

INTERVIEW TOPICS		EXPLORED FIT-AXES		
**Digital working in health care practice**				
	Views and attitudes of digitization and automation in general		Individual – Technology	
	Views and attitudes of digitization and automation in professional practice		Individual – Technology	
**Experience using digital systems and devices**				
	Changes in daily life and professional practice after the introduction of digital systems and devices		Task – Technology	
	Individual learning challenges		Individual – Technology	
	Who or what helped in getting digital skills and competencies		Individual – Technology	
	Advantages, disadvantages, and pitfalls of digital working		Individual – Task	
	Facilitators and barriers of digital working		Individual - Technology	
			Task - Technology	
			Individual – Task	
**Outlook on the future**				
	Outlook towards the future of digital working and nursing practice		Task – Technology	
	Needs and wishes to stay connected and competent on digital developments		Individual – Technology	

### The FITT-framework

**Figure 1:**
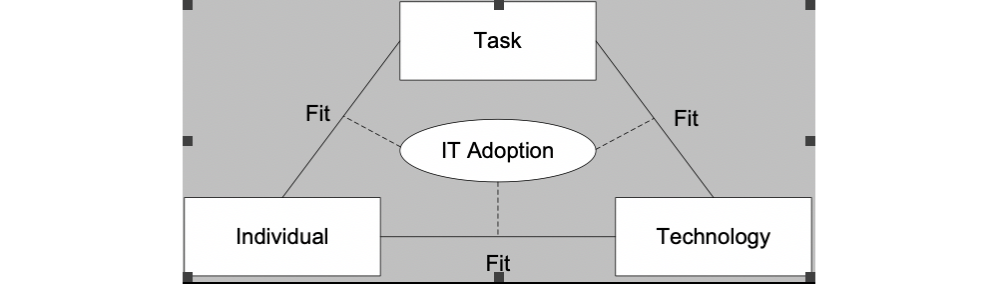
The FITT-framework of information technology (IT) adoption (Ammenwerth, 2006); FITT: fit between Individual, Task, and Technology.

FITT stands for the fit between the individual, the task, and the technology. Ammenwerth developed and used this framework to evaluate the IT adoption of nurses in clinical wards by analyzing the characteristics of the users, the tasks they had to perform, and the characteristics of the technology. The framework is shown in Figure 1. Ammenwerth describes several barriers and facilitators for each of the 3 components of the framework. A poor fit might lead to frustration and, in the end, a boycott of ICT use. According to Ammenwerth, the model can help identify and analyze reasons for ICT adoption to guide the implementation process. However, it must be noted that a stable situation on the 3 fit-axes is almost impossible due to the influences of external factors, such as changing organization structures, advances in technology, and other external factors.

### Analysis

Thematic analysis was used to analyze the data [32]. The purpose of thematic analysis was to get to know the data by reading and re-reading. First, codes were generated and overarching themes were identified. Two researchers analyzed the interviews and coded the text independently of each other by assigning conceptual labels to data [33]. The 2 researchers discussed the coding on 7 occasions and discussed discrepancies until consensus was reached. A process of identifying text fragments relating to the research question was undertaken, and 183 text fragments were marked. One researcher categorized the codes about the same parts of the research question and discussed the categorization to reach consensus. This resulted in 5 main categories and 12 subthemes (Table 2). After analyzing the last 2 interviews, no additional information was presented, and saturation was reached. The interviews were analyzed and coded using the program ATLAS.ti (version 8.0, Atlas.ti Scientific Software Development GmbH) [34]. We used the COREQ (consolidated criteria for reporting qualitative research) checklist to report the results [35,36].

**Table 2:** Main categories, subthemes, and mapping with elements of the FITT- framework; FITT: fit between Individiaul, Task, and Technology.

Category, Subtheme		Mapping with Axes of the FITT-framework
**Experience with digital working**		
	First experience and current use	Individual – Technology
	Positive and negative experiences	Individual – Task
		Task – Technology
**Perception and meaning**		
	Emotions, feelings	Individual – Technology
	Attitude toward digital working	Individual – Technology
**Barriers**		
	Lacking digital knowledge and skills	Individual – Task
	Person-related barriers	Individual – Technology
	Digital training not tailored to needs	Individual – Task
**Facilitators**		
	Acknowledgement from management	Individual – Task
	Tailored training, peer-to-peer learning	Individual – Task
	Help at hand (at work and at home)	Individual – Technology
**Future perspective**		
	General outlook to future	Individual – Technology
	Future learning needs	Individual – Task

### Ethics

The ethics committee of the Radboud University Medical Center waived the request to approve the study as it did not fall under the Medical Research Involving Human Subjects Act in the Netherlands.

## Results

### Characteristics of Participants

All 10 participants were registered nurses (RN) working in the University’s medical center. Of the 10 participants, 7 were women, 3 were men, and their mean age was 56 (median 54; range 52-63). The mean duration of employment at the hospital was 28.9 years (range 20-39). Of the 10 nurses, 7 nurses worked in a nursing ward or a daycare ward, 2 worked in the outpatient clinic, and 1 combined their nursing work with administrative tasks. Table 3 presents participant characteristics.

**Table 3:** Characteristics of participants (N=10).

Participant Characteristic			Values
Age in years, mean (median; range)			56 (54; 52-63)
**Gender, n (%)**			
	Male		3 (30)
	Female		7 (70)
**Nursing education, n (%)**			
	Diploma		4 (40)
	Bachelor		2 (20)
	Unknown		4 (40)
Years of employment at the organization, mean (median; range)			28.9 (28.9; 20-39)
**Work setting, n (%)**			
	Non-bedside		1 (10)
	Nursing ward		6 (60)
	Daycare unit		1 (10)
	Outpatient clinic		2 (20)
**Usage of devices at home, n (%)**			
	**Smartphone**		
		Used regularly^a^	9 (90)
		Used rarely^b^	0 (0)
		Never/no device^c^	1 (10)
	**PC/laptop**		
		Used regularly^a^	2 (20)
		Used rarely^b^	5 (50)
		Never/no device^c^	3 (30)
	**Tablet**		
		Used regularly^a^	2 (20)
		Used rarely^b^	2 (20)
		Never/no device^c^	6 (60)

^a^ Regularly = at least once every day.

^b^ Rarely = only now and then.

^c^ Never = owns device but never uses it; no device = not in possession of device.

### Category 1: Experience With Digital Working

The first experience of the participants with a computer or digital environment took place at work, at home, or at a study course. None of the participants had any intrinsic motivation or reason to start using digital systems or devices; this was always prompted by obligation or necessity. Almost half of them (4/10) were initially confronted with a computer or digital system during work for taking minutes, or registration of patient data, or other tasks (Participants 2, 4, 5, and 6).

All participants had a tablet, a laptop, or a computer at their disposal at home, and all of them used a computer professionally. If there were multiple digital devices available at home, there was always one that was preferred, but this preference varied from person to person. All but one participant (9/10) owned a smartphone. Most participants (7/10) mentioned that they checked their emails on a smartphone; however, some participants (3/10) did not know how to do so, and some (2/10) did not want to for reasons of principle. Most participants (7/10) used apps on their smartphones, like WhatsApp, a weather app, or a banking app. One participant that didn’t want to use a smartphone for reasons of principle said,

I don't own or want to use a smartphone. I don’t want to use apps or use the phone for all kinds of things. But I really feel under pressure that I still don't use and own a smartphone. I feel I really must defend and justify myself for that all the time.

Participant 3

Most participants (9/10) commonly used word processing as well as a search engine on the intranet or internet. The work-related applications that were mostly used were the organizational digital quality system, the EHR, and email. Rarely used or mentioned applications included Excel (used by 2 of the 10 participants), PowerPoint (used by 2 of the 10 participants), Facebook (used by 1 of the 10 participants), Skype or FaceTime (used by 2 of the 10 participants), and YouTube (used by 1 of the 10 participants). Half of the participants had typing skills.

For most of the participants (6/10), using the EHR was part of their professional practice. Reaching a basic level of proficiency with this system was not easy for them, and most still struggled. During the introduction and implementation period of the EHR, their outlook was positive, but in daily practice, they experienced the design and system functionality as user-unfriendly, time-consuming, and not supportive of the daily work process. Therefore, the EHR was experienced as a burden.

Almost all participants (9/10) said that using a computer took up valuable patient time. All were reluctant to use a computer or tablet during conversations and consultations with patients. This was perceived as an impersonal way of performing nursing care. Most of the participants (9/10) wrote their notes on paper and entered them into the system at the end of their shift rather than using the real-time documentation tools provided by the EHR. One of the participants said,

I think it’s important that the EHR is user-friendly and intuitive, so it helps me in my work as a nurse. I often feel that I’m working for the EHR system instead of the other way around.

Participant 4

However, most participants (6/10) also mentioned several potential advantages of digital working, such as no more searching for paper patient records; all patient-related information in one record; easier patient handoff; better overview of the patient’s situation, which improves patient safety; standardization of the process of nursing care; no problems in the readability of the handwriting; and being able to reach out to a colleague or a treatment team with 1 system click.

The participants said that social interaction and collegial conversations during breaks were hampered as everyone was busy with their smartphones. They shared that their own lack of digital skills and competencies regularly annoyed some colleagues. Two of the participants (2/10) said that they hoped for understanding and support from their colleagues (Participants 1 and 2). Several participants (5/10) said that age and aging played a role in dealing and working with digital systems. They experienced significant differences in digital skills and competencies compared to the new generation of “the digitally born.” Often the participants asked their younger colleagues for help in this area.

### Category 2: Perception and Meaning

The fact that digital systems play a key role in daily life and in the nursing process provoked different emotions in the participants. Most of the participants described digital working as “no fun at all,” “working in a fake world,” “stressful and annoying,” “feeling isolated,”’ “insecure,” “frustrating,” and “shameful.”

Insecurity, anxiety, and shame were feelings mentioned by most participants (7/10). These feelings contributed to a negative self-image followed by withdrawal and the desire to become invisible (Participants 1, 2, 4, 5, 6, 7, and 8). Therefore, situations that provoked these feelings were avoided, and work was left to others; participants reported feeling trapped in a situation that was difficult to change.

I notice that new digital things are constantly coming up and that change is an ongoing business. Just when I think I have reached a basic skill level, a new system or functionality is there, and I feel I must start all over again. And to protect myself from feeling too stressed and frustrated, I decide to avoid that particular situation.

Participant 10

At times, these feelings and attitudes toward digital working were overwhelming; however, participants clearly said that they were willing to learn to become more digitally skilled out of love and passion for their nursing profession and their patients.

### Category 3: Barriers

Participants mentioned several barriers to obtaining basic digital skills and competencies. There was a lack of general digital knowledge, and little or no formal digital training or education. The digital knowledge of participants was based on trial and error, and therefore, fragmented. The understanding of how ICT works was largely absent.

Using the Outlook agenda…I’ll do it, but I really don’t understand the Google and Outlook systems, and how it all works. I just don’t ‘understand it, and that makes me feel insecure and unsafe, as I don’t know what I’m doing.

Participant 3

The digital language was experienced as alienating and unfamiliar. This made it difficult for participants to take part in conversations with colleagues using digital jargon. Some participants (6/10) said that the introduction of the EHR in nursing practice was a wake-up call for them to start developing their digital skills.

Several participants (4/10) talked about personal characteristics that impacted the development of digital skills and competencies. Examples included difficulty with structuring thoughts and situations, having dyslexia, and being a practical learner rather than a theoretical learner. Some nurses found it hard to ask their colleagues for help because they did not want to take up too much of others’ time or were ashamed of doing so. The absence of digital help at home was reported as a shortcoming.

For the participants who had had formal digital training (including EHR training; 7/10), almost all of them (6/10) said that the content, form, and pacing of that education had not matched their personal learning needs and learning styles. Some of these participants reported too much information having been given over; a large learning group; the presence of young and digitally savvy colleagues; a fast learning pace; content not based on the daily work process; and lack of general information about the digital system or application that was being trained for. With digital applications that were used infrequently, feelings of incompetency increased. Also, the daily workload and work stress experienced were reported as impediments to digital learning on-the-job. Hence, new digital functionality was experienced as information overload, and it increased feelings of stress and uncertainty.

### Category 4: Facilitators

The participants reported several factors that could be helpful for “staying onboard digitally.” These factors were related to aspects of nursing management and leadership, learning conditions in class and in the workplace, peer support and help at work, and practical digital help at home.

Participants indicated that it helped if their managers were aware of their experienced difficulties in developing digital skills. Also, knowing that other colleagues experienced similar difficulties and could be talked to about it helped to reduce mental stress. Several participants (3/10) said that managers could do more by encouraging a team culture based on collegial learning, and by facilitating and emphasizing the importance of helping each other in achieving a basic digital skills level. The concept of training on-the-job was viewed as highly effective by participants. In addition, enough time to learn and to repeat skills was viewed as an essential success factor.

I talked to my manager that I got stuck […] that I had difficulties using the EHR. After that, I was linked to one of our senior nurses and EHR-superuser during day shifts, and I could ask her help on EHR topics and nursing documentation whenever I wanted. That was really helpful.

Participant 4

Participants reported several key learning elements that were crucial to their digital learning needs. These elements were a small learning group of similarly skilled people; clear instructions parsed into learning steps; a lot of recurrent rehearsal of the learning content; practice- and process-based learning; a digital learning environment for practice; and ample time allotted for practice.

### Category 5: Future Perspectives

Participants were asked how they viewed the future and what they needed to keep up with digital developments. Some of them said that concerns about the digital future were not that important to them, and some hoped that developments would pass by or stop at a certain moment (3/10; Participants 2,5,9). Some participants (2/10) were worried about their longevity in the nursing profession, and one person thought about looking for another job that was less “digitally infused” (Participant 8).

Some participants (4/10) reported that there had already been several ongoing digital developments at their workplaces but that they had reservations about using them. Some examples were patient-provider communication via the patient portal, teleconsulting, checking patient vitals with wearables (also at home), and bedside computers for patients. Some participants (5/10) believed that future hospital and nursing care would change enormously due to these developments.

At the same time, several participants (3/10) expressed a wish to be able to keep up with digital developments and to become more skillful. One participant expected improved digital skills to result in more working pleasure and satisfaction. A strategy mentioned was to use one’s practical knowledge of daily workflows to get more involved in digital development projects.

If I hear that they are going to develop something new (digital applications), I would love to be involved, just to tell how it might be practical to work with.

Participant 2

Participants also mentioned that their organizations could help by defining a basic digital skills level for employees, and by providing a monitoring and examining system for defining digital skills levels. Participants were divided in their opinions regarding their own initiative and investment in becoming digitally skilled. Some (3/10) said that it was the responsibility of employees to invest in their own digital education. But most of them (7/10) would appreciate practical help in finding the right courses tailored to their digital learning needs.

## Discussion

### Main Findings

In this paper, we present the results of an in-depth interview study to explore and identify the experiences and needs of nurses who consider themselves to be lagging in digital competency. The study was performed to deepen our understanding of the contextual and individual characteristics that affect the situation. Furthermore, we wanted to formulate recommendations for practice, management, professional education, and research.

In general, the nurses in our study can be characterized as late adopters of technology in their personal lives and in the workplace. Some of them were averse to IT and technology. In learning to use digital devices and systems (for example, an electronic patient record), the training and learning conditions were not tailored to their needs. The learning groups were too large, and the pace of learning was too fast, with insufficient time for repetition. As late adopters of technology and digital working, the participants felt that they were not given enough time to learn on-the-job, as well as insufficient support from peers. All this added to their stress, frustration, and feelings of incompetency, resulting in a tendency to postpone or avoid the use of HIT in daily nursing practice. When interpreting the research findings in the context of the 3 dimensions of the FITT-framework, there seemed to be a suboptimal fit on all 3 dimensions. Organizational interventions and measures should focus on all 3 dimensions because the balance between them affects IT adoption.

### The Context of the FITT-framework

The FITT-framework approaches IT adoption by looking at the fit between the individual, the task, and the technology. An optimal fit between the 3 framework dimensions allows for easy IT adoption. Presumably, the larger the difference between the actual fit and the planned fit, the higher number of problems that may occur during implementation of IT systems [32]. There are 3 relations of fits within this framework: (1) a fit between the individual and the technology, (2) between the individual and the task, and (3) between the task and the technology. This framework helped us categorize interview data; however, it also showed us that it is sometimes difficult to make clear distinctions between the fit dimensions, since they are not mutually exclusive but are partly intertwined and have certain overlaps[31].

In our analysis of the fit between the individual and technology, we found our group of participants to be largely incompetent with computers, electronic devices, and software. They exhibited little enthusiasm for learning about technology or systems, and this seemed to be related to the level of digitization in the participants’ personal lives. Furthermore, this group expressed reservations about utilizing an electronic device (like a computer, tablet, or smartphone) during nursing care activities. Acceptance of IT as a professional tool needs attention in this group; its use was perceived as alienating and “bad” patient-centered care (this is also a widely held opinion amongst some elderly nurses, as it goes against what they had been taught about “good nursing” [21]). This can be interpreted as a poor fit in this dimension. However, despite the intrinsic resistance to technology and systems, there is a general willingness to learn how to use basic HIT systems to keep up with the current professional and organizational developments. This presents a challenge in change management for nurse management and leadership.

In our analysis of the fit between the individual and task, we found that, although participants did mention several advantages to HIT, these advantages were not integrated into their professional competencies and daily nursing activities. The nurses struggled with changes in their work and professional collaboration with nursing colleagues due to digitalization. For example, the use of IT jargon in daily practice was reported as alienating from core nursing care language. It also negatively impacted collegiality, collaboration, and team cohesion while elevating feelings of incompetency. As the work pace is consistently high in the nursing profession, it is difficult for the participants in our study to make the required professional changes as nursing care becomes more complex and digitally infused. This issue presents a significant concern in light of the growing nursing shortages experienced in many countries all over the world [22,30]. Moreover, elderly nurses are the clinical teachers for future nursing professionals. Therefore, a possible solution lies in emphasizing the mutual learning benefits that arise from collaboration between elderly nurses and young professionals, in which the latter group teaches their mentors about digital nursing.

In our analysis of the fit between the task and technology, we found that the technology must offer sufficient functionality and performance to support a nursing task. The results of our study show a poor fit in the dimension of task-technology. For example, some participants experienced the EHR as user-unfriendly and unsuitable to the nursing task. This finding was also present in a panel study among Dutch nurses [37]. On the other hand, some participants said that after instruction and daily support from colleagues, they began to understand how the nursing process (labeled by them as a “task”) was built into the EHR. Previously accustomed to a manual method of charting and documenting, this had previously gone unrecognized and misunderstood. This is a common phenomenon among elderly nurses who must make a change from manual to digital charting [21].

### Implications for Practice

Several facilitating conditions seem to be of influence on IT adoption in this group of nurses digitally lagging behind the majority of their colleagues at their workplaces. The realization and interpretation of these conditions are the main implications for practice.

First, learning conditions and needs for this specific group differ from the average population. Carefully assembled training, tailored to learning styles and learning needs, is key. The content of any IT training must follow and reflect the daily work process and must be accompanied by learning on-the-job, peer support, and an adequate digital training and play environment. Second, the effect of social influences must not be underestimated when learning on-the-job and when environmental changes are occurring. Our cohort of nurses expressed sensitivity to social pressures to use particular technology or systems [21]. Consequently, nurse management could make use of this fact, for example, by appointing digital nurse role models who can act as digital coaches for their colleagues [21,28,29]. Third, some participants suggested that they should be involved in digital developments and innovations at early stages, as this would allow them to give advice about how technology and systems could be tailored to daily practices and the work process, and it would allow them to give input on the content of instructional learning materials. Health care provider participation in early-stage IT development has been suggested in other studies on IT adoption [9,27,30,31]. Lastly, nurse managers should be aware of their roles in improving the quality of the fit between the 3 fit dimensions, and be aware that it is not only individual attributes that are important. Mindfulness of the digital impact on the therapeutic relationship between nurses and patients is necessary [38]. This awareness makes fit management a constant, complex task for all who are involved with IT adoption and digital transformation. The patient, rather than the technology, must remain the central focus, and health care providers must be connected to IT personnel to ensure that the fit in the 3 dimensions is in scope from the very beginning of IT development. This will not only encourage the adoption of digital systems but will also improve ownership in any user group, which is necessary to optimize systems after they go live.

Following the research recommendations of our study, our hospital organization made several changes regarding the educational concept, form, and content of digital training and education. The component of training on-the-job and structured peer support has now been newly introduced and emphasized in daily practice.

### Implications for Research

This study explored the experiences, views, and needs of digitally lagging nurses with the use of in-depth interviews. The inclusion of participants was based on the observations of nurse managers and their individual conversations with their nursing personnel.

Our study identified a group of nurses who lack basic IT knowledge and skills, and consequently have a low sense of self-efficacy and self-confidence with computers and technology use. This group perceives itself to be partly invisible and sometimes ignored (although not intentionally) because they have no common face. More research regarding this group, their profile, characteristics, and learning needs is justified, with the ultimate goal of retaining them in the nursing profession and in their organizations. Expanding this study to other health care organizations, such as general hospitals, nursing homes, or primary care organizations, may corroborate our findings.

In addition, it would be worthwhile to find out if this phenomenon exists among groups of physicians and allied health care workers. A qualitative approach would be appropriate when starting to explore views, needs, and attitudes. Another line of investigation could target management and leadership of health care organizations (eg, is there any awareness regarding this group? How are these individuals identified?). Furthermore, it may be useful to expand this research to other health care organizations, like other non-university hospitals.

We did not use any quantitative instruments to measure digital competence or attitudes toward computers use. For future research, we recommend using an additional quantitative questionnaire to assess digital competency. This would complement the results from the interview data and enhance data triangulation. Another point of attention for future research is to include the age factor of participants in the analysis of quantitative results. This would allow researchers to describe relations and correlations between age and digital competency that can provide further rationale for formulating appropriate training approaches for the target group of nurses digitally lagging, as well as other applications.

There is ongoing research regarding the use and validation of the FITT-framework of Ammenwerth. A recent publication by Prgomet [39], evaluating clinicians’ use of computing devices and identifying factors affecting the optimal use of those devices, proposes an extension of the FITT-framework with the added component of “environment” to explicate the relationships between individuals, tasks, technology, and the environment in which they operate. Additional factors relating to the environment were found to affect the use of technology. These environmental attributes included, for example, physical environment, department type, organizational policies, and procedures. It would be interesting to explore these environmental aspects in further studies. In our study, we used the FITT-framework to guide the analysis of interview data. We think the framework also has the potential to be applied during HIT implementation by modifying implementation activities to the fit of the 3 axes of the model.

### Study Strengths and Limitations

One of the strengths of our study was that we succeeded in identifying and including 10 nurses digitally lagging behind. Our hospital applies a policy of regular performance interviews between nurse managers and nurses, which include evaluations on job satisfaction, performance, and professional ambitions. This contributed to the identification and inclusion of our study participants. However, the group of participants might have a limited perspective on HIT, which might have been reflected in their answers to the interview questions.

Another strength is that all participants expressed they felt safe and at ease with the interviewer to share feelings and opinions, and to speak freely about worries and experiences around IT technology and system use. Therefore, the material we collected was rich in content.

A limitation of our study is that we cannot generalize the results to other groups or to the general population, as it is a single case study in one Dutch university hospital. Despite the limited generalizability, the results of our study can inform health care organizations and care managers to provide focused attention and help for this specific target group of nurses.

### Conclusion

Our study explored the experiences and views of nurses who are digitally lagging. Although a fair amount of studies are available on factors influencing IT adoption among nurses, we could not find any studies targeting this particular group. The findings of this study support the assumption that this group of nurses could benefit from a tailored approach to digital training and education. The generic one-size-fits-all strategy does not suit their learning needs and styles. More research is needed to identify this target group in more detail in order to tailor interventions to their needs and goals of becoming competent digital nurses.

## Abbreviations

EHR: electronic health record
EMR: electronic medical records
FITT: Fit between Individuals, Task, and Technology
HIT: health information technology
ICT: information and communications technology
IT: information technology
